# Advancing health equity in the guideline enterprise

**DOI:** 10.1002/gin2.70024

**Published:** 2025-04-14

**Authors:** Andrea J. Darzi, Tamara Lotfi, Kian Torabiardakani

**Affiliations:** ^1^ Department of Health Research Methods Evidence, and Impact, McMaster University Hamilton Ontario Canada; ^2^ Department of Anesthesia McMaster University Hamilton Ontario Canada

**Keywords:** evidence‐based medicine, guideline, health equity, policy, public health, recommendations

## Abstract

- Addressing equity meaningfully helps reduce disease burden and improve health outcomes for all, including populations experiencing inequities, as highlighted in the commentaries by Persaud, Dewidar and colleagues.
- Guiding principles and tools, such as the GIN‐McMaster equity extension checklist, support the systematic consideration of equity in the guideline enterprise, ensuring recommendations remain adaptable, impactful, and responsive to evolving evidence and varying resource contexts.

Clinical and public health guidelines aim to provide evidence‐based recommendations for key practice questions. By adopting an equity lens, guidelines can help reduce unjust or avoidable disparities in achieving optimal health outcomes. In line with this mandate, we highlight two key commentaries that offer globally relevant lessons for advancing equity in the guideline enterprise.

In their contribution, Dewidar and colleagues underscore two central messages including the risks of neglecting equity considerations in guidelines and the transformative impact of addressing it meaningfully.^1^ They illustrate how the routine exclusion or marginal consideration of equity can lead to unintended consequences and perpetuate injustices in healthcare recommendations. For example, they discuss how earlier guidelines for leprosy, which failed to consider equity and human right concepts, led to harmful policies such as involuntary isolation resulting in stigma, poor outcomes, and systemic discrimination. In contrast, more recent guidelines have taken a rights‐based and equity‐oriented approach, emphasizing community integration, early diagnosis, and social inclusion through community‐based care and education. The authors showcase several WHO guidelines that effectively integrate equity principles to improve care for underserved populations such as the nonsurgical management of chronic primary low back pain which emphasizes accessibility for older adults through assistive products and infrastructure adaptations. Collectively, these examples demonstrate how equity‐informed guidelines can reduce disease burden, enhance access, and improve health outcomes for vulnerable groups. Dewidar and colleagues conclude by calling for scalable methods to support equity integration in guidelines in ways that adapt to different resource levels and evolving evidence.^1^

In a complementary commentary, Persaud emphasizes that guidelines can either champion or inadvertently undermine health equity, depending on how deliberately equity is embedded.^2^ While equity is often mentioned in passing within introductions or methods sections, it is too rarely reflected in the recommendations themselves. Persaud's commentary illustrates how such omissions can perpetuate disparities, especially when guidelines fail to prioritize interventions for disadvantaged communities or ignore settings where structural failures exist. An example of this is in emergency departments, where a lack of formal follow‐up plan can turn short‐term opioid prescriptions into long‐term misuse, particularly among patients who have limited access to primary care. By contrast, guidelines that deliberately incorporate equity, through methods such as inclusive panel recruitment, robust data collection on diverse populations, and targeted recommendations, help ensure that benefits reach those most in need. For instance, HPV self‐staffing allows individuals to screen without requiring an in‐clinic pelvic exam, which can reduce barriers like scheduling conflicts or lack of transportation. Policy level measures, such as providing financial coverage for testing or standardizing community outreach, further expand access by addressing cost burdens and geographical limitations. This approach not only improves outcomes but also enhances efficiency and builds public trust. Persaud's central message is clear: we must move beyond superficial “checkbox” equity and adopt systematic, equity‐focused strategies throughout the guideline development process.^2^

These commentaries echo the Cochrane Health Equity Thematic Group's call to action, which urges for the integration of equity considerations across all stages of research and suggests approaches to do so.^3^ To support this, Dewidar, Darzi, and colleagues, offer a clear roadmap for developers, policymakers, and clinicians by outlining seven guiding principles for embedding health equity throughout the guideline enterprise.^4^ These include defining equity in context, planning apriori for its integration at every stage, allocating adequate resources, and involving relevant interest‐holders including individuals with lived experience. Applying an equity lens during evidence synthesis and recommendation formulation ensures the needs of diverse populations are addressed, while inclusive knowledge mobilization promotes adoption and use. Ongoing evaluation further strengthens impact and accountability. These principles also underpin practical tools, such as the new Equity Checklist extension to the GIN‐McMaster Guideline Development Checklist, which supports transparent and equity‐informed guideline development.^5^

Considering equity in the guideline enterprise is both an ethical responsibility and a necessity for fostering trust in science and, ultimately, improving population health. To move beyond theoretical aspirations, guideline developers should systematically and transparently integrate equity principles throughout their work. By doing so, guidelines offer equity‐informed recommendations that fulfill the universal mandate for health as a human right that is fair, just, and attainable for all.

## AUTHOR CONTRIBUTIONS

Andrea J. Darzi and Tamara Lotfi conceptualized the editorial. Andrea J. Darzi prepared the first draft. Tamara Lotfi and Kian Torabiardakani contributed to preparing the first draft. All authors provided critically reviewed the editorial, provided edits and approved the final submission.

## CONFLICT OF INTEREST STATEMENT

Andrea J. Darzi and Tamara Lotfi are core members of the Cochrane health equity thematic group and lead authors of papers cited in this manuscript, on equity in guidelines.

## ETHICS STATEMENT

The authors have nothing to report.

## Data Availability

Not applicable, as this is an editorial. Data sharing is not applicable to this article as no new data were created or analyzed in this study.
